# A severe case of the bladder inversion treated by total cystectomy and hysterectomy with ileal conduit

**DOI:** 10.1002/iju5.12795

**Published:** 2024-12-16

**Authors:** Taro Akai, Naoki Kawamorita, Tetsuro Shiraiwa, Mahoro Watanabe, Tomonori Sato, Takuma Sato, Emi Yokoyama, Masumi Ishibashi, Zen Watanabe, Akihiro Ito

**Affiliations:** ^1^ Department of Urology Tohoku University Hospital Sendai Japan; ^2^ Department of Gynecology Tohoku University Hospital Sendai Japan

**Keywords:** bladder inversion, pelvic organ prolapse, urinary diversion

## Abstract

**Introduction:**

Female pelvic organ prolapse (POP) is considered a borderline disease between urology and gynecology and is treated by reconstructive surgery, which restores the organs to their original positions.

**Case presentation:**

An 82‐year‐old woman with bladder and uterine prolapse at POP‐Q Stage IV was treated with an indwelling catheter for voiding dysfunction. Eventually, the catheter has been often spontaneously removed when pelvic organ prolapse occurs, resulting in a diagnosis of bladder inversion. Because of bladder inversion and low bladder capacity at the time of bladder retraction, the patient underwent total cystectomy, combined with total hysterectomy, colpopexy, and posterior colporraphy, and ileal conduit.

**Conclusion:**

The cases of complete bladder and uterine prolapse combined with bladder inversion, total hysterectomy, colpopexy, posterior colporraphy, and ileal conduit were performed safely. Considering urinary function, surgery without organ preservation with urinary diversion may be an option.

**Abbreviations & Acronyms**
POP: pelvic organ prolapse
POP‐Q: POP‐quantification
QOL: quality of life
TVM: transvaginal mesh

**Keynote message**

The cases of complete bladder and uterine prolapse combined with bladder inversion with dilapidated function and bilateral hydroureteronephrosis, due to pelvic organ prolapse. Cystectomy and urethrectomy combined with total hysterectomy followed ileal conduit and posterior colporraphy were performed safely.

## Introduction

Female pelvic organ prolapse (POP) is a borderline disease between urology and gynecology, with the main symptom being a bulge in the vagina due to bladder, uterine, or rectocele prolapse. POP is considered a hernia of the pelvic region due to poor pelvic organ support. Pelvic organs are indirectly maintained in the pelvic region by the vaginal canal and POP is considered to be caused not by a problem with the organs themselves, but by damage or weakening of the ligaments, fascia, or muscles supporting the vaginal canal.^1^

POP‐quantification (POP‐Q) is used for staging to indicate the degree of ptosis. When POP is diagnosed at a high stage, surgical treatment, including hysterectomy, vaginoplasty, and vaginal retraction, is generally performed by obstetricians and gynecologists. In addition, vaginoplasty is combined with other techniques depending on the level of damage to the vaginal supporting tissues.^2^ Bladder inversion is a rare condition^3^ in which the bladder is fully extended and exits the body through the external urethral orifice.^4^

In this report, we describe a case of bladder inversion and uterine prolapse, in which a total cystectomy and hysterectomy with ileal conduit were performed.

## Case presentation

**Fig. 1:**
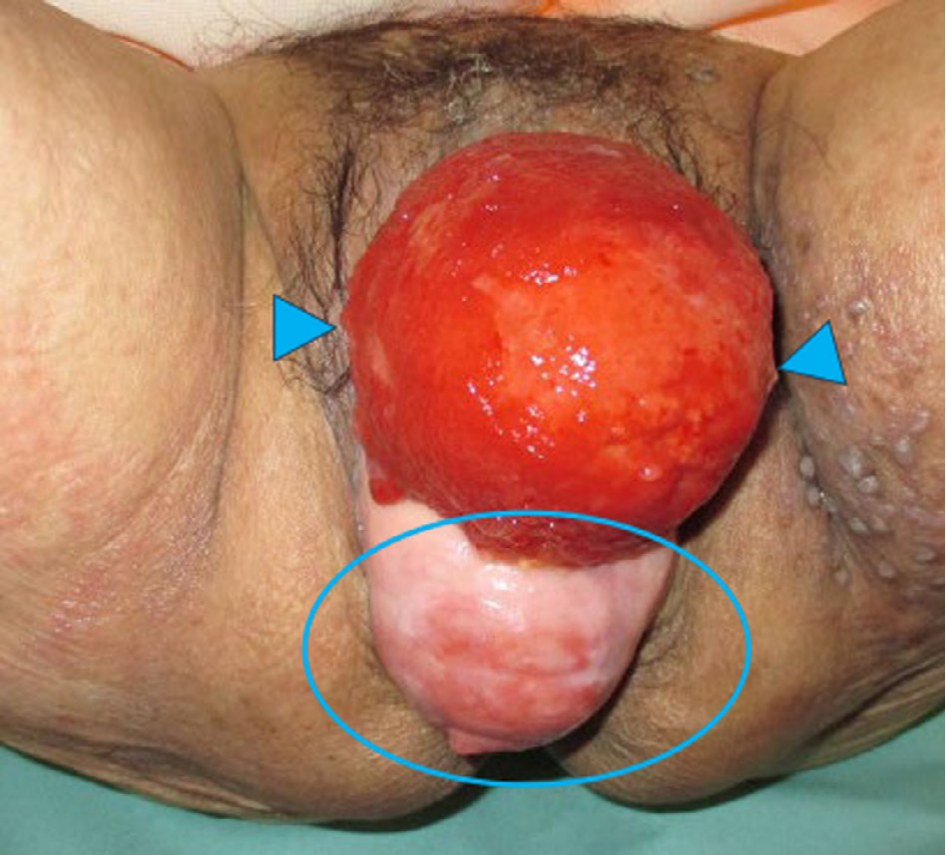
Photographs of bladder and uterine prolapse. Arrows indicate mucosa surface of the inverted bladder. Uterine prolapse circled dorsal to bladder prolapse, showing exposed vaginal wall and cervix.

**Fig. 2:**
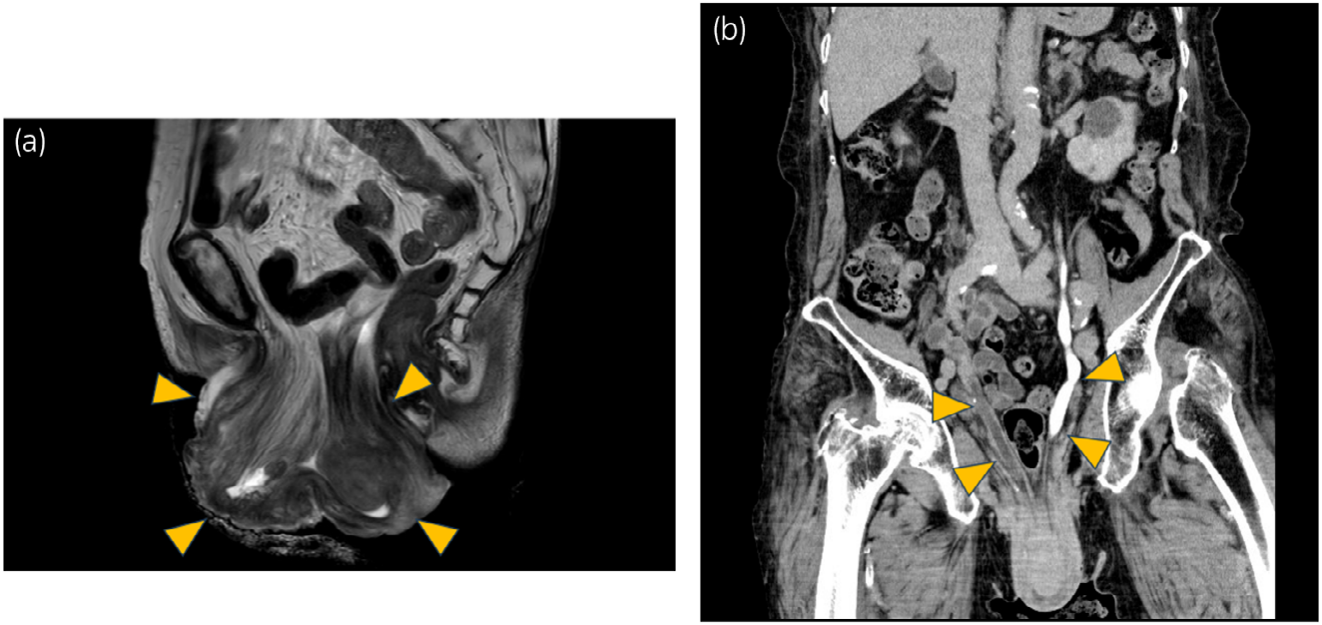
(a) MRI T2 sagittal view shows complete bladder and uterine prolapse. Arrows point to bladder and uterine prolapse. (b) The CT coronal section image shows the bilateral lower ureters were compressed at the pelvic floor and dilated proximally by the prolapsed bladder and uterus. The arrow points to the dilated ureter.

An 82‐year‐old woman presented with symptoms of POP from 20 years ago, which had been treated by inserting a pessary ring. However, the ring was spontaneously removed repeatedly, and the patient required an indwelling catheter for voiding dysfunction. The catheter was also removed spontaneously due to bladder inversion (Fig. 1). MRI and CT revealed uterine prolapse and complete bladder inversion with bladder wall thickening due to inflammation. In addition, the bilateral lower ureters were compressed by the prolapsed bladder and uterus at the pelvic floor and dilated proximally, whereas there was no obvious rectal prolapse or rectal mass (Fig. 2).

**Fig. 3:**
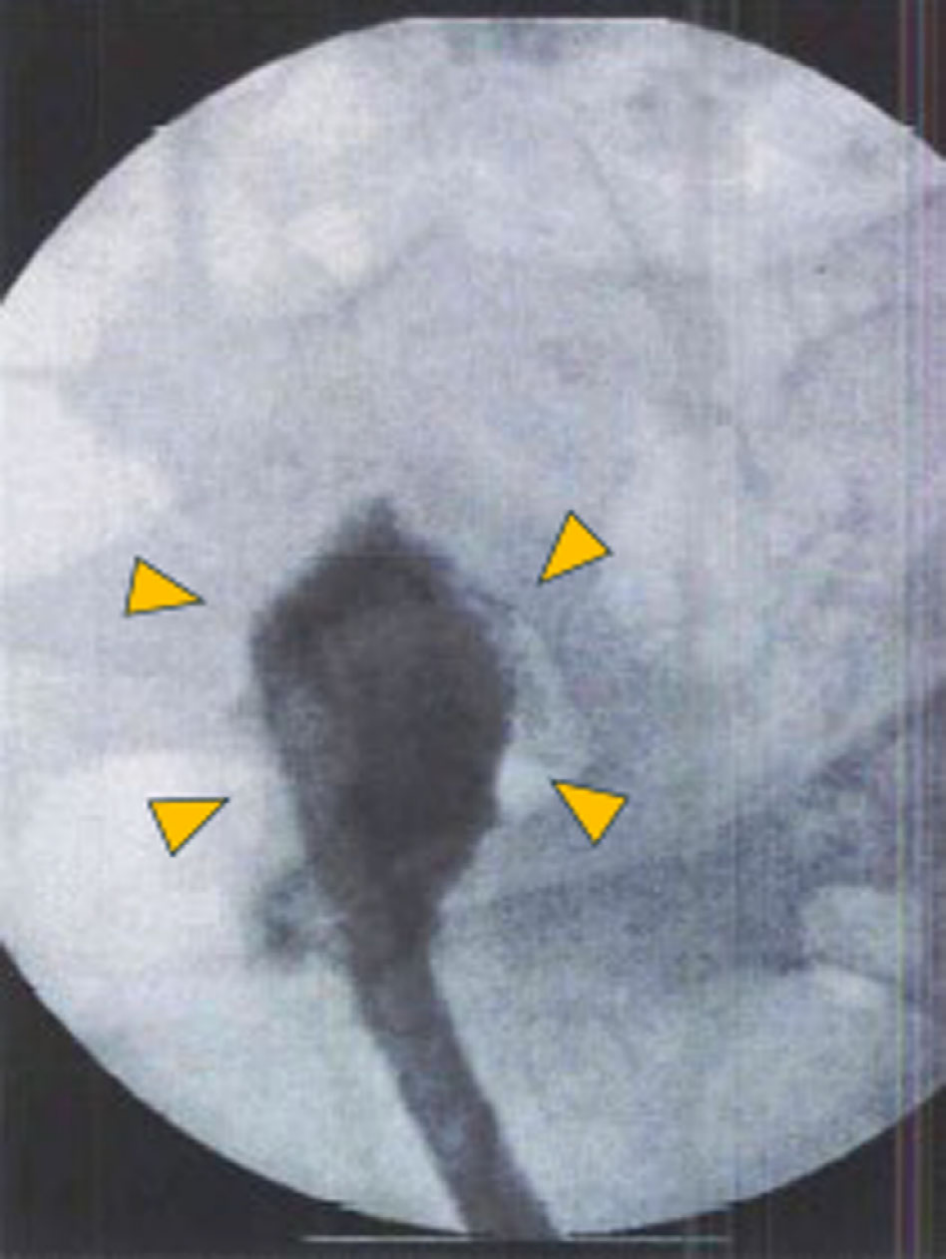
Lateral image of cystography after repair of bladder inversion is shown. Arrows indicate a contrasted bladder; ureteral reflux was not observed.

Before surgical treatment for sever POP, bladder function was evaluated by cystography under general anesthesia. The external urethral orifice was highly dilated at the time of repair and the urethral orifice opening was retracted toward the vagina. Cystography revealed a bladder capacity of approximately 40 ml and poor bladder extension; however, no bilateral ureteral reflux was observed (Fig. 3). These findings led to the decision to perform a cystectomy for bladder inversion.

Two months later, simple total cystectomy, combined with total simple hysterectomy, colpopexy (Shull procedure), and posterior colporraphy vaginal were performed jointly with gynecologists. The ileal conduit was functionally anastomosed and bilateral ureters were anastomosed using Wallace technique. Due to the prominent dilation of the external urethral orifice, it was difficult to dissect the vaginal and bladder borders, and the anterior vaginal wall was resected with a portion attached to the cystectomy side. This was because a severe urethral tear at the anterior vaginal wall occurred due to prolonged retention of the urethral catheter. The operative time was 8 h and 53 min, and blood loss was 313 ml. Histologically, the excised bladder specimen showed reactive thickening and hyperplastic changes in the urothelium lining of the lumen. However, no tumor atypia or proliferative changes were observed. Cysts and endometrial atrophy were observed in the cervix; however, there were no neoplastic changes, including bilateral adnexa. The patient was discharged on the 19th postoperative day without major complications. After discharge, POP did not recur, and the patient was satisfied with her urinary management.

## Discussion

This is the case where uterine prolapse and bladder inversion were coexisted. To evaluate bladder function, cystography was performed under general anesthesia, indicating that the bladder was a complete inversion with low compliance. Regarding surgical treatment for POP, the approach of surgery includes obliterative surgery – colpocleisis, colporrhaphy – and reconstructive surgery – sacrocolpopexy and sacrohysteropexy. In this case, the bladder capacity was small and the patient had bilateral hydroureteronephrosis due to bladder and uterine prolapse, although VUR was not confirmed on cystography. There are concerns about the urinary dysfunction affecting renal function when severe POP with bladder inversion is treated by obliterative or reconstructive surgery. Initially, the patient was considered for hysterectomy and vaginoplasty; however, it was expected to be difficult to detach the vagina from the bladder because of bladder inversion. Furthermore, the bladder capacity was significantly reduced with inflammatory changes and the patient underwent total cystectomy and hysterectomy with ileal conduit. To our knowledge, this is first case of complete bladder inversion and high‐grade POP that was treated by cystectomy.

There were few reports of treatment of cases of bladder inversion combined with POP. Tanaka reported that transvaginal mesh (TVM) was performed for uterine prolapse and manual posterior urethroplasty was performed for bladder inversion.^5^ Recently, TVM has not been recommended for surgical treatment because it often causes adverse events such as pain, infection, and erosion. Other reports of bladder inversion in the last 10 years are shown in the Table 1. In the present case of complete bladder, uterine prolapse, and bladder inversion, cystectomy and urethrotomy combined with total hysterectomy and posterior colporraphy may be an option to improve the QOL and preserve renal function while confirming the patient's wishes.

**Table 1:** Reports of bladder inversion

Author	Treatment	Uterine prolapse	Year
Decey^6^	Transvaginal bladder neck closure and cystostomy	−	2024
Ichino^3^	Robotic supracervical hysterectomy, sacrocolpopexy, bladder neck reconstruction → robotic burch colposuspension, cystopexy to the rectus fascia, bladder neck reconstruction, colpocleisis, and cystostomy	+	2022
Mae Delara^7^	Bladder neck closure, perineoplasty, and cystostomy	+	2018
Yamamichia^8^	Vaginal hysterectomy, cystopexy to the rectus fascia, colpocleisis, perineoplasty, bladder neck closure, and cystostomy	+	2018
Nishizawa^9^	Urethroplasty rectus muscle fascia sling procedure	−	2016
Tanaka^5^	Manual reduction, urethroplasty, and AP‐TVM	+	2014
Asai^4^	Manual reduction and suture the external urethral meatus	−	2014

When total cystectomy is performed, patients should undergo urinary diversion, ureterocutaneostomy, ileal conduit, or orthotopic neobladder. In previous research, there was no difference in quality of life (QOL) among patients with each urinary diversion.^10^, ^11^ Although the disadvantages of ileal conduit include pouch management, changes in cosmetic appearance, and perioperative complication risk, in this case, patients' QOL, and renal function should be prioritized. The patient was old and had restricted activities due to bladder inversion and worried about self‐catheterization after orthotopic neobladder reconstruction, while the patient had no comorbidity without hypertension. Therefore, total cystectomy with an ileal conduit was performed. There are several options to treat POP when it is stratified by the severity and organs involved. Healthcare providers should pay attention not only to the reconstruction of POP but also to morbidity and QOL.

## Conclusion

The cases of complete bladder and uterine prolapse combined with bladder inversion, total hysterectomy, colpopexy, posterior colporraphy, and ileal conduit were performed safely. Considering urinary function, surgery without organ preservation with urinary diversion may be an option.

## Author contributions

Taro Akai: Investigation; resources; writing – original draft. Naoki Kawamorita: Investigation; resources; supervision; writing – review and editing. Tetsuro Shiraiwa: Resources. Mahoro Watanabe: Resources. Tomonori Sato: Resources. Takuma Sato: Resources. Emi Yokoyama: Investigation; resources; writing – review and editing. Masumi Ishibashi: Resources. Zen Watanabe: Resources; writing – review and editing. Akihiro Ito: Supervision; writing – review and editing.

## Conflict of interest

The authors declare no conflict of interest.

## Approval of the research protocol by an Institutional Reviewer Board

Not applicable.

## Informed consent

Written informed consent was obtained from the patient for publication of this report.

## Registry and the Registration No. of the study/trial

Not applicable.
